# A Qualitative Analysis of the Commercial Broiler System, and the Links to Consumers’ Nutrition and Health, and to Environmental Sustainability: A South African Case Study

**DOI:** 10.3389/fsufs.2021.650469

**Published:** 2021-05-12

**Authors:** Kevin Queenan, Soledad Cuevas, Tafadzwanashe Mabhaudhi, Michael Chimonyo, Rob Slotow, Barbara Häsler

**Affiliations:** 1 Department of Pathobiology and Population Sciences, Veterinary Epidemiology, Economics and Public Health (VEEPH) Research Centre, Royal Veterinary College, Hertfordshire, United Kingdom; 2 Centre for Development, Environment and Policy, SOAS, London, United Kingdom; 3 Centre for Transformative Agricultural and Food Systems, School of Agricultural, Earth and Environmental Sciences, University of KwaZulu-Natal, Pietermaritzburg, South Africa; 4 School of Agricultural, Earth and Environmental Sciences, University of KwaZulu-Natal, Pietermaritzburg, South Africa; 5 School of Life Sciences, University of KwaZulu-Natal, Pietermaritzburg, South Africa; 6 Department of Genetics, Evolution and Environment, University College London, London, United Kingdom; 7 Leverhulme Centre for Integrative Research on Agriculture and Health, Royal Veterinary College, Hertfordshire, United Kingdom

**Keywords:** food system, food safety, sustainability, broilers, South Africa, Zero Hunger (SDG 2)

## Abstract

Food systems face growing challenges to meet targets of Zero Hunger (SDG 2), and South Africa is no exception given its triple burden of malnutrition, foodborne disease outbreaks, and threats of climate change to food production. Broiler meat is South Africa’s most affordable meat option, supporting household food and nutrition security. Although considered healthier and less environmentally harmful than ruminant meat, it is not without food safety risks and environmental impacts. This research aimed to present the foremost commercial broiler system narratives in South Africa, around targets of SDG 2, and to discuss key considerations for policymakers. Twenty-nine key informants and stakeholders, purposively selected to cover a wide range of opinions, participated in semi-structured interviews. Transcripts underwent a qualitative framework analysis. Results showed a highly efficient system, dominated by a small number of interlinked large-scale actors, vulnerable to competition from cheaper imports, yet pressurized to maintain high food safety and environmental impact standards, with a price-sensitive consumer base. Existing policies lack integration and enforcement capacity, and are undermined by siloed government departments, and mistrust and power struggles between public and private sectors. We propose removal of silo walls, and trust building through participatory policy development, with collaborative and transformative public-private partnerships that are designed to build capacity to deliver sustainable solutions.

## Introduction

Global food systems face substantial challenges to meet specific targets under the Zero Hunger Sustainable Development Goal (SDG 2), namely to ensure access of all people to safe, nutritious and sufficient food all year, and to build sustainable food production systems (Global Panel, 2020). Food systems in South Africa are particularly challenged, despite the country’s substantial socio-economic and political change since the end of apartheid in 1994. Childhood stunting and micronutrient deficiencies persist, whilst obesity prevalence among children and adults grows (Lundeen et al., 2016; NDoH, 2016; Bosire et al., 2020). Food safety surveillance gaps became apparent during the world’s largest outbreak of foodborne listeriosis which occurred in South Africa in 2017–18 (Thomas et al., 2020). In the past decade the country’s population grew by 17%, however food production is threatened by increasing drought frequency and climate change (Conway et al., 2015; STATS SA, 2018). COVID-19 lockdown restrictions and the pre-existing poverty levels in South Africa, have increased food insecurity risks for the poorest households through job losses, poor access, and unstable (national and international) supply chains (Perez-Escamilla et al., 2020).

South Africa has the highest meat consumption per capita in Africa, with a growing demand for poultry meat, showing an increase of 132% between 1995 and 2015 (DSEA, 2016; Ritchie and Roser, 2019). Broiler meat remains the most affordable meat option and plays an important role in household food and nutrition security, and in the South African food system (GAIN, 2015; McHiza et al., 2015). Whilst considered healthier than red meat by some (Bouvard et al., 2015; Godfray et al., 2018), its impact on consumers’ health is complex, with portion sizes and preparation methods playing an important role (Schönfeldt et al., 2014).

The South African commercial^1^ poultry (broiler and layer) industry is the single largest contributor to agricultural related GDP, and an exemplar of the country’s commercial livestock sector, with its production systems and efficiency comparable with other global intensive production systems (DAFF, 2018; SAPA, 2018b). The broiler value chain is dominated by a small number of large-scale commercial producers, who produce >75% of national production (SAPA, 2018b). The largest are vertically integrated, with formal marketing and distribution networks, involving a few large supermarket chains, and quick-service (fast-food) restaurants, including international franchises (Louw et al., 2017; Ncube, 2018).

Foodborne disease (FBD) presents a greater risk for individuals affected by poverty, malnutrition, reduced immunity (HIV/AIDS), or chronic diseases like tuberculosis (TB), all of which are over-represented in South African society (Lund and O’Brien, 2011; Thomas et al., 2020). Foodborne disease outbreaks in South Africa are commonly associated with *Salmonella* spp., *Clostridium perfringens*, *Bacillus cereus*, *Shigella* spp., *Listeria monocytogenes*, and *Escherichia coli* (Shonhiwa et al., 2018). The main pathogens found on poultry meat include *Staphylococcus aureus*, *Camplylobacter* spp., *Listeria monocytogenes*, and *Salmonella* spp. (Goncalves-Tenorio et al., 2018). South Africa’s recent foodborne listeriosis outbreak was traced to a factory producing a low-cost processed meat product containing broiler mechanically deboned meat (MDM) and meat from other livestock sources (Thomas et al., 2020).

Broiler meat is regarded by some as the least environmentally damaging meat option (Willett et al., 2019). However, the rising broiler meat consumption levels brings into question its environmental sustainability. Whilst intensive production has limited direct impacts on land use, it has indirect environmental impacts through its total dependence on cereal based feed (Skunca et al., 2018). Only 11% of South Africa’s land area is suitable for cropping, and 3% is used for cereal production (for human and livestock consumption), most of which is rainfall dependent and vulnerable to climate change (Conway et al., 2015; Trading Economics, 2020). Feed costs comprise 65–70% of intensive production costs, and the broiler industry consumes ∼2.8 million tons of feed, made mostly from yellow maize and soybean (SAPA, 2018a). South Africa, whilst generally self-sufficient in maize (outside of drought years), imports 60% of the poultry industry’s soybean needs, leaving an environmental impact in the exporting country (SAPA, 2018a). Other environmental impacts of production include water use (in cleaning, and processing), energy use in environmentally controlled housing, and management of waste (Skunca et al., 2018).

Our research forms part of the Sustainable and Healthy Food Systems (SHEFS) programme, which aims to provide policymakers with novel, interdisciplinary evidence to define future food system policies that deliver nutritious and healthy foods, in an environmentally sustainable, and socially equitable manner. The aim is therefore well-aligned with meeting several targets within the SDG of Zero Hunger. To date, many experts have expressed opinions about the unsustainability of food systems, but most have come from distinct and diverse disciplines (Béné et al., 2019). The result has been a range of distinct conceptual frameworks characterized by the links between agriculture and nutrition (Nicholson et al., 2020). However, complex food system challenges call for an interdisciplinary, One health and whole systems approach to gain a better understanding of the system’s complexity and interconnectedness (Zhang et al., 2018; Garcia et al., 2020). Consequently, our work is framed within a wider “food systems approach” as described by van Berkum et al. (2018); a theoretical framework which recognizes the various elements within the system and the relationships between them. Preceding this study, we conducted a systems analysis of the livestock-derived food system in South Africa and developed a conceptual system dynamics model, which highlighted the importance of broilers (Queenan et al., 2020). With broiler system as our focus, we used a food systems lens to conduct a qualitative analysis aimed at answering the broad question: How does the commercial broiler system in South Africa contribute to meeting the SDG 2 targets that relate to consumers’ health and nutrition, food safety, and the environment, and what barriers and opportunities exist for change? We present the main narratives based on the perspectives of a wide range of stakeholders and key informants within the broiler system, and discuss challenges and key areas for policymakers’ consideration. These results will be used, in part, to develop a system dynamics model of the South African broiler system. This will demonstrate feedbacks, reinforcing and balancing loops, and system archetypes, and will aim to provide evidence for policymakers on the various interrelated outcomes of specific policy scenarios.

## Methods

This qualitative research, using semi-structured interviews, was preceded by our systemic analysis and development of a conceptual system dynamics model of the livestock-derived food system in South Africa. This deepened our understanding of the complexity of livestock-derived food system, the importance of broilers within the agricultural sector, and as the source of the most affordable and commonly consumed meat. This in-depth knowledge was a useful foundation and guide in the formulation of the interview topics and questions (Rabionet, 2011). It also determined our focus on the commercial broiler sector given its contribution to overall production. Research on the small-scale and emerging producers was conducted in parallel and is under review with this journal.

### Data Collection

Twenty-two semi-structured interviews were conducted in South Africa with 29 participants (13F, 16M), either individually, or at most in pairs within the same institution. Participants were selected purposively from authors (whose gray and academic literature was reviewed in previous work), professional networks, and online searches. Additional individuals were identified through multiple entry-point snowballing and “horizontal networking” (Geddes et al., 2017). Our selection aimed to represent a wide cross-section from the commercial broiler system, to ensure diversity of perspectives, and to capture common themes within the divergence (Patton, 1999; Creswell, 2014). Participants included those representative of large, medium and small-scale commercial producers, importers, input providers (feeds and medicines), animal health service suppliers (veterinary practitioners, both public and private), human health laboratory specialists, academics and researchers in natural resources, economics, animal health, human health and nutrition, and representatives from the broiler producer association, non-governmental organizations, and government departments and agencies (see Appendix 1).

The interview questions were piloted among colleagues experienced in conducting qualitative research to provide an opportunity for feedback, revision and improvement (Turner, 2010). Interviews were conducted jointly by two researchers in English and face-to-face where possible. When people were unavailable or could not be visited, then phone or online virtual meetings were held. One interviewer was South African, which was beneficial to interpret local vernacular and lingua franca expressions (Qu and Dumay, 2011). After outlining the study’s aims and explaining data usage and confidentiality, interviewees were given an opportunity to ask questions before providing written consent, which included permission for audio recording.

Open questions were asked that focussed on interviewees’ opinions of recent broiler industry trends, the role of the industry in consumers’ food security and nutrition, food safety issues, the impact of industry on the environment (and vice versa), and significant past and current policies that impact the industry. Audio recordings were transcribed using an independent South African transcribing service, which allowed for more accurate transcription of local accents.

Ethics approval for this research was gained from the Royal Veterinary College’s (University of London) Social Science Ethical Review Board (URN SR2018-1624), and the University of KwaZulu-Natal’s Human and Social Sciences Research Ethics Committee (HSS/0235/018D).

### Data Analysis

Data was analyzed using a framework analysis, which is an approach specifically designed for the analysis of policy relevant research, where the objectives are commonly established by researchers in advance, and data analysis is informed by a priori reasoning (Pope et al., 2000; Ward et al., 2013; Parkinson et al., 2016). The methodology is also most suited to multidisciplinary research and the thematic analysis of semi-structured interview transcripts, which by nature are less heterogeneous than informal, unstructured interviews (Qu and Dumay, 2011; Gale et al., 2013). This approach also aligned with our research aim, since it addresses a variety and combination of research questions, concerning the context, purpose, evaluation, and future strategies around the subject of interest (Ritchie and Spencer, 1994).

The framework analysis used the five steps as developed by Ritchie and Spencer (1994), and described in Parkinson et al. (2016). These are (i) Familiarization with data: This was accomplished by the interviewing researchers (KQ, SC) reading the transcripts, and discussing emerging categories for the framework. (ii) Identifying framework categories: These were kept within the boundaries of the research questions and interview structure, yet allowed for addition of emerging issues. (iii) Indexing/coding: Transcripts were color coded according to framework categories. (iv) Charting: Coded data were transferred directly into the framework in Microsoft^®^ Excel. Some framework categories were revised and disaggregated further. (v) Mapping and Interpretation: This involved summarizing the categorized framework data, mapping out topics, aligning the data interpretation with the research questions, finding patterns and concepts, and identifying themes. Turner (2010) recommended that interviews be combined with other forms of data collection to fill gaps in the analysis. Therefore, we identified peer-reviewed and gray literature via Google searches, and used them to provide background context to themes, and as methods-triangulation to enhance quality of analysis (Patton, 1999).

## Results

Within the boundaries of consumer health, nutrition, and the environmental sustainability of the system, the following themes were developed from the qualitative framework analysis, and are presented with additional background context information. Whilst policy elements are referred to within most themes, a separate policy theme is presented at the end.

### Dominance of Integrated and Large-Scale Producers in a Dichotomous System

In 2018, South Africa slaughtered an estimated 18 million locally produced broiler birds per week, yielding 1.8 million tons of broiler meat in the year (SAPA, 2018a). Local production is dominated by two companies that jointly contribute ∼46% of total production in almost equal shares, whilst a further five producers supply 29% (DAFF, 2017). The two largest producers are vertically integrated, controlling their own input supplies (including breeder flocks, feed mills), abattoirs, processing plants, and marketing and wholesale outlets. Integrators also use contract growers to whom they supply chicks, feed, vaccines, advice on production and disease control, and veterinary and laboratory services. The integrator guarantees a market and price for the contractors’ grown stock, which is recognized as an advantage by contractors, together with cash-flow savings on the major inputs (feed and chicks).

- We don’t have to outlay the food and chicks. And you get a guaranteed market; we don’t have to do any marketing. If you’ve grown 250,000 birds and then have to look for a market; no thank you (190606_001).

At the other extreme, small-scale farmers supply an estimated 20–25% of the broiler meat produced, some through formal market channels, whilst the majority concentrate on the informal live bird market. The middle ground between large and small-scale producers has become increasingly vacant.

- They (medium-scale producers) really didn’t have a spot in the whole system. All my (medium-scale) guys are basically packed up and either joined the corporates or changed to different production, from broilers to broiler breeders or things like that. We have seen more smaller guys coming up all over. I think it’s at the expense of the mid-sized guys (190618_001).

### Local Producers and Importers Fight for Their Market Share

Recent trade agreements (relaxing import tariffs) have resulted in imported poultry supplementing (or competing with) local production. Interviewees specifically referenced the United States (US) African Growth and Opportunity Act (AGOA) from 2000. Other agreements included the EU’s Trade, Development and Cooperation Agreement. Tariff relaxations were primarily on bone-in frozen portions, but these were increased again in 2018 in response to the significant increase in imports. In 2018, imports peaked at 566,210 tons, an increase of 44% since 2014 (SAPA, 2019), and ∼95% of this was chicken (almost all was frozen, and of broiler origin). The top three product categories and approximate proportions were, (i) 45% “bone-in portions”, (ii) 33.3% mechanically deboned meat (MDM), and (iii) 8.2% clean offal (gizzards, livers, heads, necks, feet, but excluding intestines) (SAPA, 2018c, 2019).

Interviewees within the local production industry claimed that import volumes equate to the presence of a new integrator in the market. Importers disagreed arguing that broiler import figures were not just frozen broiler meat, but included MDM. They stated that MDM should be considered as a separate commodity to broiler meat, and that the import volumes of actual imported broiler meat and offal are significantly less than the 25–30% that is often quoted. Importers also highlighted that cuts used in the exporting countries to produce MDM (necks and backs), are consumed in their original form by South African consumers. As a result, there was little or no local production of MDM, and importers therefore claimed that imported MDM was vital for the local processed meat industry, which provided around 15,000 jobs, and offered a low-priced animal-sourced protein for lower socio-economic status consumers.

- About 15% (of broiler meat) consumption is provided by imports. We (importers) don’t include MDM when we do our calculations, because we say, it’s not directly competing with chicken in the supermarket or a butchery shelf. MDM, it’s sold as polony, or some form of a cooked, smoked sausage. Those products are sold as a competitive product in the market, but it’s not chicken, and it’s not sold as chicken. It might ultimately land up competing, but it’s competing as a different product category against chicken (190517_002).

### Unlevel Playing Field for Importers and Local Producers; Winners, and Losers

Before the democratically elected government of 1994, local commercial producers were supported by government subsidies, and protected by import tariffs (Hendriks, 2014). Local industry interviewees were fiercely critical of the lack of support and protection offered by current government, suggesting that the local industry will collapse if imports are not controlled.

- If you look at all the countries in Africa where there aren’t local industries anymore, it was decimated by imports. Ghana, Mozambique, Nigeria, Angola. And it’s simply, chicken comes in cheap until the local industry is dead, and then chicken prices go up. The countries with no industry, relying on Brazil predominately for imports, it (chicken) is very expensive, the same price as red meat in South Africa (190515_001).

It was reported that government was of the opinion that local production could not meet the increased demand for broiler meat, and they viewed imports as a means to improve food security for the lower socio-economic status consumers. However, local producers claimed they have no incentive to invest and increase production because they cannot compete with overseas producers that receive subsidies from their own governments.

- We are getting this (imported chicken) from countries where farmers are heavily subsidized, so obviously the South African producer is fighting a battle with somebody who are not coming on equal ground (190624_002).

Breast meat is a consumer-favored and highly profitable cut in high socio-economic markets internationally, whilst the remaining bone-in cuts are less favored, with offal considered a by-product (BFAP, 2016). By contrast, it was reported that almost half of South African consumers prefer bone-in cuts (sold in mixed-portion bags of individually quick frozen (IQF) pieces), and many also enjoy consuming all parts of the bird, including all forms of offal (including intestines). Therefore, importing bone-in cuts and offal products is seen as a market opportunity by importers. Several interviewees recognized that imports challenge the local industry’s efficiency, whilst others suggested that local production would not be able to fill the gap left by imports, should tariffs be applied to reduce them.

- It is a local industry that is underperforming. I think it’s an opportunity that your importer would see (190517_003).
- Definitely, in terms of food security, it (imported chicken) is a priority. If we raise our tariffs, and then we’re going to have shortages, and the SA industry cannot supply enough for the market (190624_001).

Several participants felt that the rise in imports have affected small-scale producers that sell live birds the least. In contrast, they perceived medium-sized producers and emerging commercial farmers as the least able to adapt, and the hardest hit. Integrators have reportedly reacted by taking steps to improve efficiencies, expand or consolidate their market options, or widen or revise their product range. Some integrators reportedly sold off land assets and closed old houses to improve margins, but not without consequent job losses. They opened new, more energy-efficient houses on new, more strategically located sites, closer to the markets, cereal production and feed mills. In addition, they diversified risks, by increasing the proportion of their production delivered through contract growers. High-end quick-service restaurants (QSR) were targeted by integrators as a lucrative and expanding market. An interviewee, with previous experience in QSR chains, reported that imported broiler cuts did not meet their strict traceability requirements and smaller size specifications (for which QSR pay a premium). Local integrators reportedly dominate this QSR market because they were more likely, than small or medium sized independent producers, to consistently meet these requirements.

- (Integrators) want in the QSR and the value-added sector. They don’t want whole bird business… So those spec birds are usually birds that have to meet a specific weight and a specific size, specific kind of uniformity… That’s where the guys can get more money for their product. (190625_001)

### Tension Between Government, Local Producers, and Importers; A Search for Agreement

Evidence of tensions between the local industry and government was noted by several interviewees, with suggestions that the historic race inequality in commercial farm ownership, was an undercurrent for the lack of government support.

- We perceive Government as hostile rather than friendly. Large scale commercial poultry production (in South Africa) is white monopoly capital, and the ANC Government is not in favor of that. So that relationship, there is a certain tension in that, and you are not sure what you are going to get from Government in terms of protection (190520_001).

Interviewees from government ministries, importers, and observers of the industry, offered other perspectives on imports. These included signs of indifference from government.

- Industry players will always have something to complain about. They will say “We are being unfairly outcompeted” by whoever, and “They’re dumping their products here” (190624_001).

The complexity of trade agreements around tariffs, specifically those liked to AGOA, was also acknowledged.

- The US uses all sorts of other trade agreements to strong-arm us into accepting their poultry dumping, you know, tariffs on other goods. It’s a lot of commodities which are being exchanged between these countries, minerals and things, so it’s many different sectors. I think it’s a very complex. So, it’s a dirty game and I think the government plays it as best they can (190521_001).

In recognition of the market tensions developing, between local and imported products, the “Poultry Masterplan” was agreed and initiated late in 2019. This was a facilitated collaboration between the Departments of Trade and Industry (DTI), of Agriculture, Land Reform and Rural Development (DALRRD) (previously DAFF), the South African Poultry Association (SAPA), and the South African Association of Meat Importers and Exporters (SAAMIE). It was described as “a strategy approach, that brings government and industry and other stakeholders (together) on how to develop and grow an industry” (190726_001), and it aims to invest in local industry growth (including incorporating smaller producers), protect local production from cheap imports and rising feed costs, and improve sanitary requirement for export markets (Details of the masterplan are yet to be released publicly).

### Provision of Affordable Broiler-Product Options for Consumers

Despite significant socio-economic progress, South Africa remains one of the most unequal countries in the world (World Bank, 2018). The growth of middle classes and urbanization, typical of growing economies, has driven a rise in meat consumption (specifically poultry meat), however 43% of the population are moderately food insecure (Ritchie and Roser, 2018; FAO, 2020). Several interviewees reported that the bulk of consumers chose chicken-based products largely on price.

- Food security in SA is not about availability of food, it’s about the cost of food (190515_001).
- Things like chicken necks, chicken stomachs, chicken livers, chicken hearts, are the only affordable animal protein for many of our population. And I think chicken is the one staple that has kept animal protein on most consumers’ plates, even for the poorest consumers (190517_002).

It was stated that price increases would preclude even the low-cost broiler meat options for lowest-income consumers, and that there was little room for value chain actors to increase their prices in response to increased costs, without the risk of their customers switching to cheaper, alternative, non-animal-sourced products.

- I mean when people were battling (economically), they were spending money on potatoes, cabbages, mielie (maize) meal and things like that, and moving away from meat altogether (190618_001).

### Food Safety Standards and Surveillance Lack Consistency and Capacity, in a Population With a High Proportion of Individuals Vulnerable to Foodborne Disease

Broiler meat and processed products are linked to many foodborne pathogens such as *Campylobacter* spp., non-typhiodal serotypes of *Salmonella enterica* (NTS), *Listeria monocytogenes*, and Shiga toxin-producing strains of *Escherichia coli* (Heredia and Garcia, 2018). A significant proportion of the South African population is particularly vulnerable to FBD, given the prevalence of TB, HIV/AIDS, malnutrition and poverty (Thomas et al., 2020).

- We also have a huge population that is susceptible, especially for non-typhi Salmonella, in our HIV positive populations, and a lot of under-fives. So, we’ve got the very highly susceptible populations (190517_001).

Public and veterinary health experts reported that laboratory services are contained within a two-tiered system, of private and government run facilities, and apart from notifiable diseases, there is no obligation for private laboratories to share data. Similarly, although there are ∼260 government run National Health Laboratory Service laboratories, only five process food samples. The majority of food testing is conducted in private laboratories, linked to private food processors and retailers. It was the opinion of a government laboratory interviewee that private laboratories’ reluctance to share data, often citing client confidentiality as the reason, potentially delayed the identification of the source of the 2017–2018 listeriosis outbreak. Counter-intuitively, their reluctance to share data, post-listeriosis, reportedly increased rather than decreased.

- Often if you are dealing with outbreaks in the private sector, or when you try to do trace-backs, they use commercial or private food-testing laboratories, and then you can’t access that data or isolates. And especially now in the wake of the listeriosis outbreak, the clients, who send their samples to private food laboratories, insist they don’t want their data or isolates shared at all, because of litigation (190517_001).

Several participants made the point that public food safety surveillance lacks capacity and integration, and is fragmented across the Department of Health (DoH), DTI, and DALRRD, and their agencies, each with different roles and with some overlap. Integrators and value chain actors involved in animal production, slaughter, and processing of resultant meat products, are regulated by DALRRD. However, the DoH regulates those trading in meat, which includes importers. Whilst the DoH’s Food Safety Directorate is responsible for policy, the operational aspects are devolved to municipalities, and the latter’s capacity depends on local budgets, which may vary widely.

- The Department of Agriculture (now DALRRD), ourselves (DTI), and also Health, now and then, we talk about what we need is one food agency, so that everything can fall under the food agency. But to get it off the ground is a problem because you have all these people who are doing different things. So, some people might feel that maybe that means that’s the end of their jobs (190624_001).
- It’s a bit of a mish-mash, right. Department of Health does a lot of the work on the food safety, but Department of Agriculture Forestry and Fishery (now DALRRD) also has that, and they have directorates that are meant to deal with it. But you know, it’s always the integration that’s an issue, as well as the structures; they inhibit the sort of collaboration (190607_001).

In the broiler system, commercial producers, formal retailers, QSR chains, and large-scale importers have reputational and financial incentives for maintaining high levels of food safety, often setting industry standards above that required by government, and managing their own monitoring systems, using in-house or private laboratories.

- Obviously, they have a huge amount of reputational damage if anything happens. That’s what happened with listeriosis, Tiger Foods was massively hammered. I suspect they have all ramped up their food safety protocols since listeriosis (190528_003).

A sub-theme of government distrust, and lack of support felt by large-scale local industry actors, was repeated by several participants. Large-scale actors felt they were overly targeted, whilst small operators went unchecked, and likewise local producers felt that they were under greater scrutiny than importers, and that there was inadequate capacity to police the latter.

- Domestic producers say because they are nearby, it’s easy to go and visit them, that the agencies, when it comes to enforcement, they enforce a lot more for them. I guess sometimes it’s just in issue of manpower, that when products are coming in through the ports, maybe there is not enough people to check the containers and the information is not as clearly descriptive of what’s contained and all that. So, in some cases, ja (yes), products come in that should have been not in, but ja (yes), it’s not so easy (190624_001).
- There are 1,500 abattoirs credited to export here. What should happen is that the importing country (veterinary inspector) visits the particular slaughterhouse, approves the slaughterhouse and the South African government vet signs responsibility over to the exporting country vet, and he must sign off and ensure that they comply with our import regulation. We haven’t visited one abattoir in 10 years. So there is no control there, nothing whatsoever (190515_001).

With respect to surveillance integration and data sharing, there is however optimism from relevant stakeholders around the plans to establish the National Public Health Institute of South Africa (NAPHISA), which is “taking a One Health perspective, so hope(fully) it will improve things” (190607_001). It will be benchmarked against the US’s Centre for Disease Control and Prevention, and aims to “use formal agreements with public and private health and food testing labs to refer isolates and share data” (190517_001), and thereby provide integrated and coordinated disease surveillance and evaluation of interventions.

### Food Safety: A Lower Priority Than Price for Consumers

It was reported that food safety, for consumers of lower socio-economic status, remains a lower priority than price. For some “any food you can get is food and to survive they would eat anything” (190528_003). This is despite the listeriosis outbreak being linked to a processed meat product, commonly bought by lower-income consumers.

- The lower-end consumer, they became very aware of it (food safety) too, which I think probably they wouldn’t have been as sensitive, prior to listeriosis, about things like food safety. I think prior to that most people have been fairly blasé about it. I mean, going back not too very long ago, really poor people, people on the edge of poverty and below, were actually digging up poultry mortality pits to consume the mortality (carcass). That’s how desperate people have been for food. If you’re prepared to do that, you know, food safety is not one of the big items on your radar (190528_003).
- A huge part of the consumer wants the cheapest product. He does not care where it comes from. He does not care if there is an expiry date on it, or what brining it has got. He only looks at the price. He can afford this chicken and that is what is going to go on his table (190515_002).

Whilst the listeriosis outbreak was traced to a product retailed through the formal value chain, there was concern expressed by the stakeholder in foodborne disease surveillance, that food safety in the small-scale and informal value chain is neglected, and needs improvement. Basic knowledge, from animal health and biosecurity, to slaughter and food hygiene, is apparently lacking with many of the informal value chain actors. They are also less able to absorb costs of implementation, and there is little incentive if they are neither monitored, nor experience repercussions. Implementation costs of international food safety standards across all systems, may push smaller actors out of business, or result in costs being transferred to consumers.

- Compliance costs on food safety are high. We do need to look at strengthening our food safety systems and using new technologies; but if we are going to do that, you know it comes at a cost. There is a huge health benefit, but someone would have to bear the costs (190726_001).
- Chicken is a mass product, so at the end of the day, I think the consumer wants something that is safe, however they define it, but at the best possible price (190520_001).

### The Industry Is Vulnerable to Climate Change Through the Impacts of Droughts on the Feed Sector, Water Quality, and Avian Influenza Risk

Feed is the greatest variable cost for producers, and as a result, the greatest concerns regarding climate change were the implications of droughts and weather variability on local cereal yields, the need to import raw materials for feed, and the associated fluctuations in feed quality and costs.

- We used to have droughts every 10 years, now we have droughts every 3–4 years, so climate change is having a massive impact; if you compromise on your grain sector then you’re in trouble (190726_001).

With only 11% of the land surface in South Africa being suitable for cropping, the total area suitable for maize and soybean is reportedly shrinking due to climate change (Conway et al., 2015;DSEA, 2016).

- Climate change is making a lot of these areas a lot more marginal. I mean Free State and that, have been hammered in recent years with droughts, and you know those predictions (of climate change), it’s going to become more of an issue (190607_002).

Droughts also impacted on the quality and reliability of water from municipal and private boreholes sources, with pollution and contamination by raw sewerage being reported. As a result, large scale producers and processors have invested in independent water supplies and in-house purification systems, and have been driven to maximize water efficiencies. Climate change was also mentioned in relation to changing patterns in wild bird migration, and increased risk of avian influenza spill-over into poultry flocks.

- Other impacts of climate change are around the increased incidents of avian influenza. You know, we are seeing certain diseases arriving here that weren’t ever present. So we’ve got no doubt that migratory patterns of birds are changing. All of these things, particularly around climate change, are throwing new challenges (190726_001).

### The Industry’s Impact on the Environment Is of Less Concern to Them Than the Impact of the Environment on the Industry

The environmental impacts of the industry seem of less concern to the main actors in the commercial system. Some recognition was given to water use, waste management (including bird mortalities), energy use in maintaining stable conditions in environmentally controlled houses, and land use for cereal crops. However, compliance by large-scale producers to the environmental impact assessment (EIA) regulations, as set out in the government’s National Environmental Management Act (1989) (DEA, 1998), and Environmental Impact Assessment Regulations (2014) (DEA, 2014), was considered by them to be adequate in mitigating environmental impacts of the industry. They also criticized the EIA process as being costly, bureaucratic, and an example of a comprehensive, yet over-zealous legal framework, with more bureaucracy than enforcement capacity.

- The other thing that will also continue to put pressure on our market, will be the whole approach, the pressure that we have in adhering to certain environmental factors, and the scale of things that we can do, as well as how and when, what has been policed and enforced (190625_001).
- The EIA regulations have become increasingly more bureaucratic, to the point that a lot of farmers will just try to avoid it, either by trying to fall below the threshold or just carrying on and pretending they don’t know about it (190607_002).

Waste management was considered by local producers to be less of a concerning issue than in the industrial systems in Europe, due to South Africa’s relatively large land space, and dryer climatic conditions. Waste from production houses was seen to have value, commonly used by crop farmers as manure, either directly onto cropland, or composted to reduce nitrogen levels. Disposal of bird mortalities are regulated through environmental legislations, and by DALRRD (with regards to contagious animal diseases like avian influenza), however, some chicken mortalities were reportedly disposed of via captive-crocodile farms that produce crocodile skins and meat.

- We have large land areas and relatively small production. The bulk of manure is used on maize lands, fed to livestock, and yes some of it probably ends up in water causing a bit of water pollution, but because of the larger spaces and much drier climate that Europe, its impact is probably mitigated (190520_001).

Fossil-fuel based heating and ventilation systems are used in environmentally controlled housing. Gas is considered too expensive for heating, and with electricity supplies being unreliable, it was estimated that 80% of producers are dependent on coal for heating. It was also recognized that there is room for improvement, following US production systems that use litter-based, biogas generating facilities, but these were considered currently cost prohibitive in South Africa.

It was recognized by some, that the limited arable land in South Africa, which is constrained primarily by rainfall patterns and water supply, was under competition for use to grow cereals for human consumption, and for animal feeds. Additional conflicts were reported between conservation and agriculture, as pressure increased to convert more natural grasslands into arable land, and also concerns as mining encroached into arable lands.

### Policy Environment Is Rich, but Poor in Governance, Integration, and Implementation

A recent review of South African food policies highlighted gaps, contradictions, and poor intersectoral coordination with regards to policy development and implementation (Boatemaa et al., 2018). Governance within South Africa’s food system has been criticized for failing to deliver much needed transformative change, primarily due to the siloed nature of government departments, and a tension between the often ambitious objectives and the constraints that exist for those tasked to implement them (Termeer et al., 2018). Furthermore, food system problem-framing has lacked a systems approach, with solutions often favoring a focus on agricultural production and food security (Drimie and Ruysenaar, 2010). Food safety governance and policies (reviewed after the listeriosis outbreak of 2017–2018), were found to be poorly coordinated and fragmented across three government departments, with the lack of implementation capacity being filled by private self-regulation in the formal sector but with gaps remaining in the unregulated informal sector (Boatemaa et al., 2019). Within the commercial broiler system, existing policies were considered by interviewees to be adequate, if not overly detailed and complex. Their main concern was the failing in policy application, and a lack of implementation capacity, which was also noted in the literature (Fourie, 2018; Queenan et al., 2020).

- You could manage within existing policies for instance in South Africa to drive this, but you need the will, and you need the people, the right people with the correct technical know-how as well as the will, you know, the will across the board, with existing industry, with government, you need the right support for the programmes. We write beautiful plans in South Africa; we fail to implement (190517_003).
- What we do is, we put a lot of effort into finding an excellent law, developing it and putting it through parliament, and then we discover that we have no capacity to enforce it or even implement it (190520_001).
- Because, the concept in this country is that we are quite over regulated. We have got a very comprehensive legal framework, but the application and the implementation of it is a real challenge (190607_002).

Some reported that specific policy and regulations were impractical, unenforceable, and needed reviewing. Examples were given concerning government departments’ responses to abattoir surveillance findings, regulations around thawing and refreezing imported meat, and thresholds set for requiring an EIA.

- Working with the Department of Agriculture is very challenging, because they don’t always come up with rational policies to be honest. They had a very limited capacity. Poor understanding of the epidemiology. I mean running a poultry abattoir, it’s quite a sophisticated operation, and then you have to work with technical people, who have no understanding or clearly very limited understanding of what’s at stake. I mean you cannot shut down an abattoir for 2 days, you are going to have X hundred thousand birds all, you know, the system just doesn’t allow for that (190520_001).
- If you want to thaw out meat and resell it, the carcass core temperature and the carcass surface temperature is not allowed to go above 7 degrees. That is the law and probably the same law in the US or UK. So it’s very difficult to police. You literally need to stand there and wait for the guy to thaw out for 24 h to prove it. So, the legislation is badly written and therefore now they make it difficult to implement, so they’ve decided not to implement that (190515_001).
- The (EIA) trigger for poultry is the number of birds, but it doesn’t really consider where in relation to the sensitive environment or something. A lot of developments in the rural areas do not go through the same processes, and the big corporates and commercial farmers are forced to, they get hammered if they don’t follow due process, but it’s a kind of free for all in the communal areas (190607_002).

## Discussion

This research aimed to investigate the commercial broiler system within the wider food system in South Africa, and its contribution to meeting the targets of SDG 2 that relate to consumers’ health and nutrition, food safety, and the environment. We analyzed the perspectives of a wide range of stakeholders within the system, and highlighted several key areas for policymakers to consider, when developing food system policies aimed at the targets of SDG 2. These included the imbalance of power within the system, tension and mistrust between local producers, importers and government, the price sensitivity of consumers, gaps in food safety surveillance, vulnerability to climate change, and the fragmented nature of policy with shortfalls in implementation.

Before specific policy issues within the broiler system are dealt with by policymakers, the lack of trust and imbalance of power between the main government and industry players, which was highlighted by multiple participants, must be addressed. Commercial and larger-scale farming was historically restricted to farmers from the white minority population, supported by British colonial rule, and later by the Nationalist government’s policy of apartheid, which ended in 1994 (Tihanyi and Robinson, 2011). The post-apartheid government’s agricultural reforms removed parastatal marketing boards and financial structures that supported commercial farmers, and replaced them with Black Economic Empowerment (BEE) policies and land reform, which are ongoing (Tihanyi and Robinson, 2011; O’Laughlin et al., 2013). However, the commercial broiler industry still has notable barriers to entry (primarily prerequisites of substantial capital and technical skill capacity), and has been slow to change from its well-established historic roots (Hall, 2004). This political backdrop is considered by existing commercial producers to influence government policy, which is perceived as unsupportive on several levels. Most notable are policies around import tariffs, and implementation of regulations pertaining to animal health, food safety and environmental impacts, which are perceived as biased against powerful large-scale local producers. Large-scale producers and importers take an approach of self-regulation and self-determination, arguably in response to the government’s lack of capacity and support, but potentially contributing to the existing tension and power struggle. Such perceived inequalities by private industry support a vicious cycle of ongoing mistrust and lack of interest in collaboration.

Broiler import tariffs are part of complex international trade deals, which typically involve trade-offs with more valuable commodities. Global power imbalances present additional barriers for South African government negotiators when attempting to meet trade objectives on food and nutrition security (Greenberg et al., 2017). Nevertheless, there is evidence from elsewhere in Africa, that cheap broiler imports outcompete local production on price, eroding local production and the associated feed industries (Dieye et al., 2007; Banson et al., 2015). Import surges also undermine local producer confidence to invest in business expansion (Greenberg et al., 2017). However, downward pressure on prices from imports was reported by some participants as potentially contributing to food and nutrient security, especially to the lower income consumers. Whilst the local industry is calling for more government support and tighter import restrictions, little was said on how capacity to cover shortfalls would be developed. An opportunity exists for the commercial industry to support the marginalized emerging and smaller-scale producers, through mentorship and skills development. Government initiatives to support this would strengthen the delivery of more inclusive agricultural development.

Social inequality persists in South Africa, and racial disparity has arguably been replaced by an economic one, as evidenced by the country’s top ranked position in the world for income inequality (World Bank, 2018). Similarly, South African healthcare, historically polarized based on race, is now largely determined by socio-economic status. Approximately 16% of the population use private healthcare, mostly facilitated by private health insurance, whilst the remainder (84%) use public health facilities (Naidoo, 2012). Disparate systems were also noted to exist in surveillance of food safety and foodborne diseases, being either government or privately funded and controlled. Weaknesses and inequalities arising from these systems were unveiled by the listeriosis outbreak, and more recently by the SARS-CoV-2 pandemic (Nwosu and Oyenubi, 2021). Further analysis, as the pandemic unfolds, will provide evidence that can drive accelerated policy reform in this area.

Socio-economic lines also divide provision of veterinary healthcare for livestock. Private veterinarians service the wider commercial farming sector. Integrators go a step further, employing veterinarians to provide in-house health and laboratory services. By contrast, state veterinarians, who rely heavily on animal health technicians, provide capacity-stretched services to the non-commercial sector (Fermet-Quinet et al., 2014). The World Organisation for Animal Health (OIE) recently stated that, with an ever-increasing demand for national veterinary services to improve livestock productivity and food safety standards, both private and public veterinary services will need to look for opportunities to collaborate and expand capacity (Thevasegayam et al., 2017). Such collaboration is needed in South Africa to address inequalities and capacity gaps in animal health services, and similarly in human healthcare, and food safety surveillance.

Attempts to swiftly resolve the foodborne listeriosis outbreak were reported by some participants to have been undermined by the lack of capacity within, and integration between government departments, and a lack of co-operation between private and public surveillance. More joined-up and consistent policy on food safety and FBD surveillance is needed that encompasses both locally produced and imported boiler products. Solving such complex policy issues requires horizontal collaboration between several government departments and non-governmental stakeholders, but are hampered by the typically narrow-focussed, siloed structures of organizations (Urban, 2018). Therefore, the establishment of an overarching body responsible for food safety is urgently required, along the lines of the European Food Safety Authority or the Canadian Institute of Food Safety, as examples. Improving food safety surveillance and compliance will come at a cost, which according to our interviewees, is unlikely to be absorbed by consumers. Food prices and income are known to strongly affect consumers’ food choices (Muhammad et al., 2017). Burger et al. (2015) reported that the lowest quartile of South African households was extremely price sensitive in respect to food choices, especially meat, whilst van Wyk and Dlamini (2018) showed a 1% increase in food prices in South Africa would reduce household welfare by 21.3%, with the greatest impact on household food security of the poor. Underinvestment in food safety and foodborne diseases surveillance is often due to an underestimation of the burden (Grace, 2015). The burden and potential benefits of interventions are best appreciated through a whole-system or One Health lens, which spotlights the links between animal health, productivity and food safety, with environmental sustainability and ecosystem health, and with human health and nutrition (Häsler et al., 2017; Garcia et al., 2020). Policymakers should therefore review the role of both public and private actors in food safety, with the costs associated with improving food safety being positioned alongside public healthcare savings, nutritional benefits, and the cost of reputation loss for private enterprises.

Public-private partnerships (PPP) offer an opportunity to help address the disparities apparent on many levels within the broiler system. They are defined as “a long-term contract between a private party and a government agency, for providing a public asset or service, in which the private party bears significant risk and management responsibility” (World Bank, 2014). South Africa has a strong track record of using PPP, with examples in health, energy, water and waste, and often linked to government’s BEE policies (Arimoro, 2018). However, PPP should go beyond the simple procurement or transactional level, and move toward collaborative and transformative models, which involve a joint commitment from all parties to deliver mutually agreed policies, and develop sustainable capacity to deliver durable solutions with business returns (Galiere et al., 2019). Such partnerships could be used to initiate steps toward rebuilding public-private trust, and achieving win-win outcomes (Roehrich et al., 2014).

A topic that interviewees perceived less relevant, yet remains key to reaching SDG 2 targets, was the industry’s impact on the environment. A more complete understanding of broiler meat’s environmental impact can be gained through a product lifecycle analysis, which considers all elements from farm inputs through to consumer’s plate (ISO, 2006). Such an analysis of intensively produced broiler meat showed the environmental impact of feed as the most critical (Skunca et al., 2018). In the South African context, the environmental impact of both imported and locally produced feed should be considered when seeking areas for improvement or mitigation. Broiler waste (and its use as manure) causes ammonia, methane and nitrous oxide emissions, soil acidification, and potential contamination of water sources with phosphates and nitrates through runoff (Belloir et al., 2017). The health implications of nitrate pollution of drinking water through agriculture, is receiving increasing attention of late (Ward et al., 2018). Policy to further mitigate environmental impacts must consider any additional costs to actors in the system, in the light of the already tight margins of producers. With the exception of consumers within the highest income bracket, most South Africans, the bulk of whom prioritized price even before food safety, would similarly be unlikely to pay more for a product’s environmental sustainability credentials. In the absence of substantial consumer driven demand for environmental sustainability, government incentives and support will be key in polices aimed at hitting the environmental targets of SDG 2.

The role of low-cost broiler meat and products in obesity was not mentioned by participants during the interviews, with the exception of the nutritionist. Participants instead focussed on broiler meat being an affordable source of animal protein and micronutrients. Although it is also considered healthier than red meat, in terms of non-communicable diseases, the preparation method, and the portion size is crucial when considering its nutritional impact (Schönfeldt et al., 2014). “Big Food” i.e., food distributed through large-scale food companies and retailers, is dominant in the South African food environment (especially for broiler meat) (Igumbor et al., 2012). Whilst a discussion of the drivers of food choice is beyond the scope of this work, Big Food is implicated in driving unhealthy eating habits and obesity through increasing the marketing, affordability and accessibility of more processed and “junk” food, creating a shift from traditional to modern diets (Igumbor et al., 2012). National food consumption data in South Africa requires updating, to help unravel the link between broiler meat and the extremes of over and under-nutrition, before developing policy to address this complex issue. The dominance of Big Food may provide a leverage opportunity to employ wide-reaching change for the better.

Complex food system challenges require a whole system approach, with wide multi-stakeholder representation (Zhang et al., 2018). The latter improves stakeholders’ understanding of the system, and their appreciation of others’ perspectives, facilitating a shift in their mental models (Ruegg et al., 2018; Zhang et al., 2018). Our research was restricted by not all invitees being willing to participate (not uncommon for high level actors within large institutions), but despite this, our sample-size exceeded that deemed appropriate in the literature (Guest et al., 2016). Nevertheless, by using a wider food system lens in our approach, our results spotlight the interconnectedness of elements within the broiler system, and how changes would bring about tradeoffs and implications elsewhere, with particular reference to balancing imports and local industry growth to maintain food security, and to who bears to the cost of improving food safety and environmental impact mitigation. Future work should aim to be more inclusive, transdisciplinary and participatory when seeking to engage with stakeholders, which could form a foundation on which to begin building trust, a pre-requisite for finding mutually agreeable solutions and engaging in PPP (Roehrich et al., 2014).

Within the diversity of opinions and perspective of participants, it was clear that the commercial broiler system will have a key role to play in reaching SDG 2 targets, given the relative affordability, popularity and increasing consumption rate of broiler meat compared to other meat. In addition, a consistent message from participants was one that called for policy change and refinement; the need for policy to be more pragmatic, and to be backed up sufficient implementation capacity, with even-handed enforcement. Much work remains to address inequalities and mistrust between stakeholders in the broiler system in South Africa, before it can move toward ending malnutrition and ensuring access for all to safe, healthy, nutritious, and sustainably produced food, all year round.

## Conclusions

Our research explored the commercial broiler system as part of the wider food system in South Africa, and analyzed the diverse perspectives of industry stakeholders around topics of consumers’ health and nutrition, food safety, and the environment. We identified strong disparate opinions on the role of imports and of local production in meeting the growing demand for broiler meat. We outlined the governance, and policy implications and associated challenges, many of which share commonality with rapidly developing commercial broiler systems elsewhere (e.g., India and Pakistan). Our approach also provides a reference for researchers exploring comparable broiler systems that are experiencing substantial change and similar policy challenges.

A lack of integrated, pragmatic policy, and the capacity to enforce it systemically, echoed throughout this study. Siloed government departments, protection of private business interests, recent political history, and related elements of tension and mistrust, were identified as the main stumbling blocks to change at the level of key system actors. These issues were driven by divergent narratives on imports, perceived bias in enforcing regulations, and a power imbalance between private and public sectors. At the consumer level, their price sensitivity was a barrier to considering issues of quality, safety or socio-environmental externalities of food. Equally, interviewees implied that middle and low-income consumers’ price driven choices, and affordability barriers for the lowest income groups, meant a low willingness to pay for higher food safety assurances on broiler meat, and consumers potentially opting for cheaper processed meat products or non-animal sourced foods as alternatives. Although the role of broiler meat in the triple burden of malnutrition received little attention, it needs to be better understood, given the rising consumption levels. Whilst the industry recognized the threat of climate change on feed and profitability, less attention was given to the impact of the broiler system on the environment, both locally and internationally, and this needs to be examined further.

This study highlighted the complexity of the commercial broiler system of South Africa, and revealed the policy and governance challenges within it. We propose the removal of relevant interdepartmental walls in government, and a clearer definition of public roles and responsibilities within the system. Future policy focused research should aim to be participatory and inclusive of stakeholders from across the system. This will allow a greater appreciation of different stakeholders’ perspectives, and facilitate the process of trust building, which is a prerequisite to move toward building integrated, inclusive and mutually agreed policies. Once such policies have been developed, collaborative and transformative models of PPP can build the capacity for the delivery of sustainable solutions.

## Supplementary Material

Appendix 1

## Data Availability

The raw data supporting the conclusions of this article will be made available by the authors, without undue reservation.

## Footnotes

1 “Commercial” is a termused in South Africa to describe privately owned, business orientated large-scale farming operations, with a high level of formal market engagement. These were, but are no longer, exclusively owned by white farmers, but the latter still remain over-represented.
