# Supplementary material for: A Qualitative Analysis of the Commercial Broiler System, and the Links to Consumers’ Nutrition and Health, and to Environmental Sustainability: A South African Case Study
Source: Front Sustain Food Syst. Author manuscript; Available in PMC 2023 Sep 7. (PMC7615038; doi:10.3389/fsufs.2021.650469)
Supplement: Appendix 1 [file EMS187261-supplement-Appendix_1.docx]

## Appendix 1

|  | **Participant Code** | **Participating individual or organisation** | **Interviewee numbers** |
| --- | --- | --- | --- |
| 1 | 190515_001 | Representative of broiler industry | 1 |
| 2 | 190515_002 | Private animal healthcare provider and advice centre | 2 |
| 3 | 190517_001 | Human communicable disease control expert | 1 |
| 4 | 190517_002 | Importer | 1 |
| 5 | 190517_003 | Independent agency for control of poultry disease (Public-private partnership) | 1 |
| 6 | 190520_001 | Private veterinarian, poultry specialist | 1 |
| 7 | 190521_001 | Poultry health academic researcher | 1 |
| 8 | 190521_002 | Nutrition and food security academic researcher | 1 |
| 9 | 190528_003 | Head of training centre for poultry management at all scales | 1 |
| 10 | 190603_001 | Poultry science academic researchers | 2 |
| 11 | 190606_001 | Contract farmer for integrated producer (large-scale 250,000 birds/cycle (actual figure)) | 1 |
| 12 | 190607_001 | Researcher in antimicrobial resistance | 1 |
| 13 | 190607_002 | Researchers conducting applied research in the environment and natural resources | 2 |
| 14 | 190613_001 | Independent emerging commercial farmer (small-scale (40,000 birds/cycle as per SAPA definition) | 1 |
| 15 | 190614_001 | Ex-contract grower for integrator (medium-scale) | 1 |
| 16 | 190618_001 | Independent feed mill supplying large and small-scale farmers | 2 |
| 17 | 190618_002 | Provincial state veterinarian | 1 |
| 18 | 190624_001 | Government Department of Trade and Industry | 2 |
| 19 | 190624_002 | Government Department of Agriculture | 1 |
| 20 | 190625_001 | Independent feed mill, with ex-logistics manager of international fast-food franchise | 2 |
| 21 | 190726_001 | Not for profit, economic research institute | 1 |
| 22 | 190730_001 | Free range large-scale integrated producer | 2 |
